# Roles of Asp179 and Glu270 in ADP-Ribosylation of Actin by *Clostridium perfringens* Iota Toxin

**DOI:** 10.1371/journal.pone.0145708

**Published:** 2015-12-29

**Authors:** Alexander Belyy, Irina Tabakova, Alexander E. Lang, Thomas Jank, Yury Belyi, Klaus Aktories

**Affiliations:** 1 Gamaleya Research Institute, Moscow, Russia; 2 Faculty of Biology, Lomonosov Moscow State University, Moscow, Russia; 3 Institute for Experimental and Clinical Pharmacology and Toxicology, University of Freiburg, Freiburg, Germany; 4 Freiburg Institute for Advanced Studies (FRIAS), University of Freiburg, Freiburg, Germany; 5 Centre for Biological Signalling Studies (BIOSS), University of Freiburg, Freiburg, Germany; Institute Pasteur, FRANCE

## Abstract

*Clostridium perfringens* iota toxin is a binary toxin composed of the enzymatically active component Ia and receptor binding component Ib. Ia is an ADP-ribosyltransferase, which modifies Arg177 of actin. The previously determined crystal structure of the actin-Ia complex suggested involvement of Asp179 of actin in the ADP-ribosylation reaction. To gain more insights into the structural requirements of actin to serve as a substrate for toxin-catalyzed ADP-ribosylation, we engineered *Saccharomyces cerevisiae* strains, in which wild type actin was replaced by actin variants with substitutions in residues located on the Ia-actin interface. Expression of the actin mutant Arg177Lys resulted in complete resistance towards Ia. Actin mutation of Asp179 did not change Ia-induced ADP-ribosylation and growth inhibition of *S*. *cerevisiae*. By contrast, substitution of Glu270 of actin inhibited the toxic action of Ia and the ADP-ribosylation of actin. *In vitro* transcribed/translated human β-actin confirmed the crucial role of Glu270 in ADP-ribosylation of actin by Ia.

## Introduction

A group of Gram-positive microorganisms, including *Clostridium botulinum*, *C*. *difficile*, *C*. *perfringens*, *C*. *spiroforme*, and *Bacillus cereus*, produces actin-ADP-ribosylating “binary toxins”. These toxins consist of a binding component, which is involved in toxin up-take, and an enzymatically active component, which harbors ADP-ribosyltransferase activity. Both components are secreted as separated proteins [1,2]. The binding component of binary toxins is proteolytically activated, forms heptamers, and binds to membrane receptors of eukaryotic target cells. After docking of the enzyme component to the binding component, the toxin complex is endocytosed [1,3–7]. In an acidic endosomal compartment, the toxin heptamer inserts into membranes and forms a pore, which allows the translocation of the enzyme component into the cytosol.

In the cytosol, the enzyme component ADP-ribosylates monomeric G-actin at Arg177. Thereby, actin polymerization is blocked by steric hindrance [8,9]. Moreover, ADP-ribosylated actin acts as a capping protein at the barbed ends of F-actin to inhibit polymerization of non-modified actin molecules [10]. Altogether, these effects cause depolymerization of the actin cytoskeleton, major morphological changes of target cells and inhibition of motile and signaling functions, which depend on the intact actin cytoskeleton [1,2,11]. Moreover, it was shown recently that destruction of the actin cytoskeleton by toxin-catalyzed ADP-ribosylation induces formation of microtubule-based protrusions, which are involved in increased adhesion of bacteria [12].

Arg177 of actin, which is modified by the toxins, is essential for actin functions. This residue is changed in the zebrafish cardiofunk actin mutation (Arg177His), which causes major defects in embryonic cardiac development and function [13]. Biochemical studies with yeast actin showed that the Arg177His mutation results in increased critical concentration, a prolonged nucleation phase and a faster elongation process of actin polymerization, suggesting increased fragmentation of actin filaments [14].

In a recent study, the crystal structure of the enzyme component (Ia) of iota toxin was analyzed in complex with its protein substrate skeletal muscle α-actin at high resolution [15,16]. Several structural “snapshots” were determined showing the actin-Ia complex in the course of the ADP-ribosylation reaction. Moreover, essential amino acid residues of the ADP-ribosyltransferase and of actin involved in catalysis were identified. These data resulted in a strain-alleviation model of ADP-ribosylation of actin by iota toxin [15].

However, in spite of recent progress in the understanding of the ADP-ribosylation of actin catalyzed by Ia, the available data lack biochemical confirmation of the proposed molecular mechanism of the reaction. In previous studies, site-directed mutagenesis was restricted to the iota toxin molecule [17,18] but amino acids of actin, suggested to be crucial for the toxin-catalyzed ADP-ribosylation reaction, were not analyzed so far.

To gain more insights into the Ia-actin interactions, we used the yeast *Saccharomyces cerevisiae* as a model. In amino acid sequence, yeast actin is ~90 and 87% identical with mammalian β/γ- and skeletal muscle actin, respectively [19]. Moreover, yeast actin is readily modified by iota toxin. We substituted yeast wild-type actin by site-specific amino acid variants in *S*. *cerevisiae* and analyzed the functional role of specific amino acid residues proposed to be crucial for iota toxin-catalyzed ADP-ribosylation of actin, using engineered yeast cells. Our findings indicate that Asp179 of actin, which was proposed to play an important role in toxin-induced ADP-ribosylation [15,16], is not essential for ADP-ribosylation by iota toxin. However, we identified Glu270 as an essential amino acid for modification by iota toxin.

## Results

### Actin engineering in *S*. *cerevisiae*

Budding yeast *S*. *cerevisiae* is a single-cell eukaryotic organism, which is used for studies on molecular mechanisms of bacterial virulence factors [20]. We employed the yeast model to engineer *S*. *cerevisae* by substituting wild type actin with its sequence variants, containing site-specific changes of amino acids suggested to play a role in toxin-induced ADP-ribosylation.

**Fig 1:**
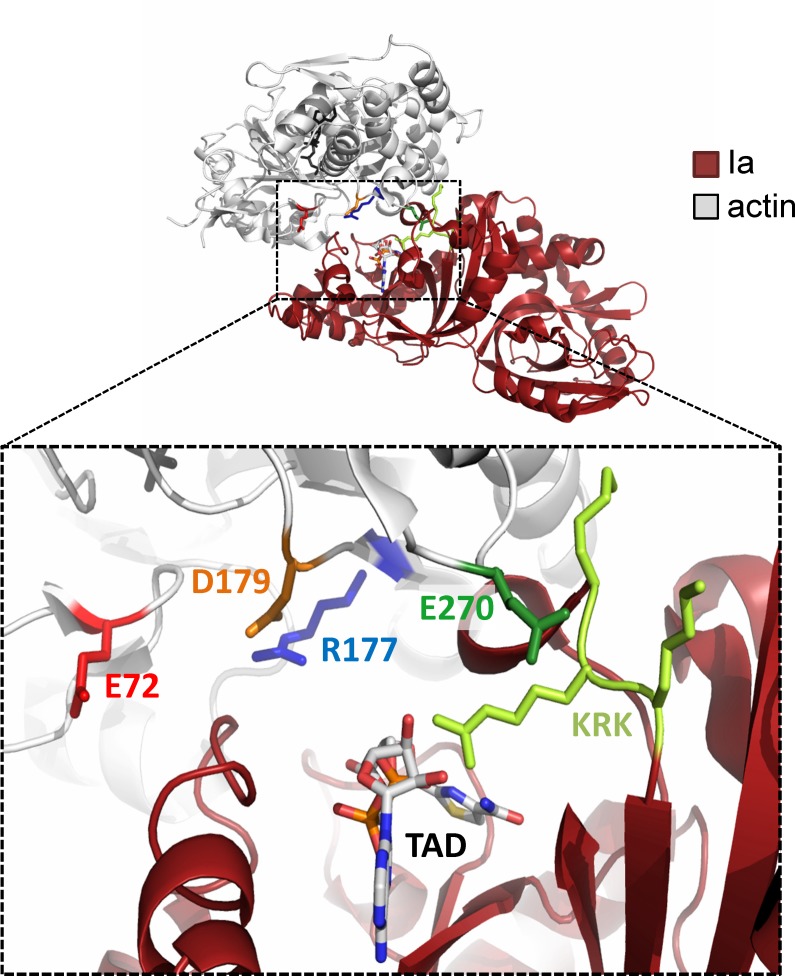
Structural representation of actin-Ia interaction. Upper panel, general view of Ia interaction with actin (pdb code 3BUZ). Lower panel, detailed representation of the region around R177 of actin. Amino acid residues R177, D179, E270, E72 of actin, K351R352K353 motif of Ia and the nonhydrolyzable NAD analog TAD (β-thiazole-4-carboxamide adenine dinucleotide) are shown as sticks. Images were prepared using PyMOL (www.pymol.org).

At first, we engineered a haploid *S*. *cerevisiae* strain with an inactivated *ACT1* gene and with functional *ACT1* on an *URA3*-bearing plasmid (*S*. *cerevisiae act1*::*LEU2* + p*ACT1* [*URA3*], see S1 Results and S1 and S2 Figs). This strain was transformed with plasmids coding for different actin variants and a *HIS3* auxotrophic marker. Subsequently, we used 5-FOA to eliminate the *URA3-*containing plasmid, resulting in cells, which contained only the *HIS3*-plasmid encoding the actin variants [21]. Deduced from the crystal structure of the Ia-actin complex [15], we studied actin mutations of Asp179 and Glu270, which are located close to the ADP-ribose acceptor Arg177 and at the interface between Ia and actin (Fig 1).

**Fig 2:**
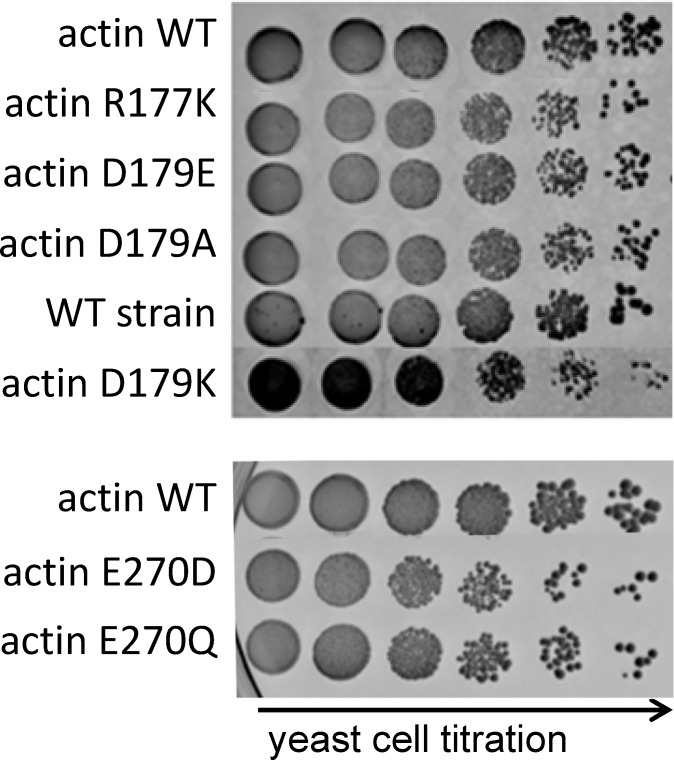
Analyses of agar growth phenotypes of *S*. *cerevisiae* containing different actin variants. Five-fold serial dilutions of yeast cultures were spotted onto YPD agar. Plates were incubated for 3–4 days at 30°C. Actin variants produced by the corresponding *S*. *cerevisiae* strains are shown on the left. Control yeast strain representing wild type *S*. *cerevisiae* is indicated as “WT strain”.

Yeast cells, containing Arg177 and Asp179 actin variants, were viable and did not demonstrate major growth defects during cultivation on YPD medium (Fig 2). This result indicates that the amino acid substitutions did not affect functional activity of the proteins under these conditions. Exchange of Glu270 (E270D and E270Q) resulted in minimally reduced growth of *S*. *cerevisiae*.

### Toxicity of Ia for *S*. *cerevisiae*

**Fig 3:**
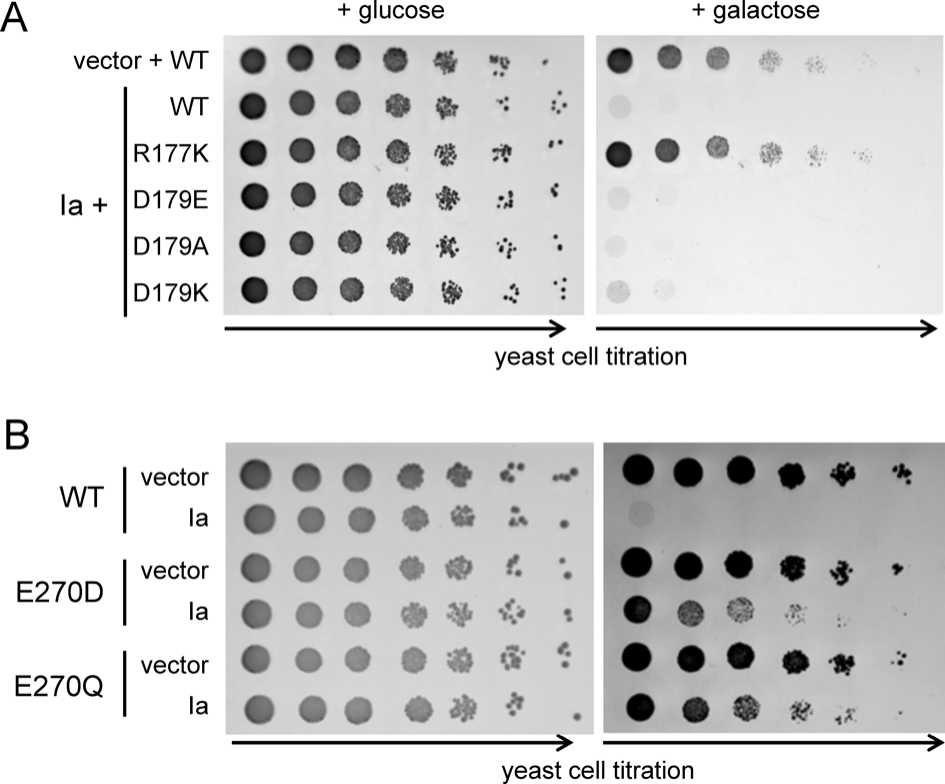
Susceptibility of *S*. *cerevisiae* containing different actin variants towards iota toxin component Ia. Yeast strains containing wild type actin or actin variants with substitutions R177K, D179E, D179A, and D179K (panel A) or E270D and E270Q (panel B) were transformed with the vector alone or the Ia-expressing plasmid, and were analyzed by the drop-test under *ia*-repressing (glucose) or -inducing (galactose) conditions. Plates were incubated for 3–4 days at 30°C.

Next, we expressed the Ia-coding gene in yeast, containing different actin variants and studied the toxin-provoked growth phenotypes of *S*. *cerevisiae*. As shown in Fig 3A, induction of the Ia-coding sequence by cultivation of transformed yeast on galactose-containing agar medium was accompanied by dramatic reduction in viability of yeasts containing wild type and Asp179Ala/Glu actin variants. In contrast, replacement of the ADP-ribose acceptor amino acid Arg177 by lysine rescued the corresponding *S*. *cerevisiae* strain from intracellularly expressed Ia. Yeast cells producing Ia in *S*. *cerevisiae* actin-Asp179Lys background demonstrated a minimally increased survival rate in comparison to the wild type *S*. *cerevisiae*. Surprisingly, we observed that Glu270Asp and Glu270Gln actin variants were largely resistant to the expression of Ia (Fig 3B).

**Fig 4:**
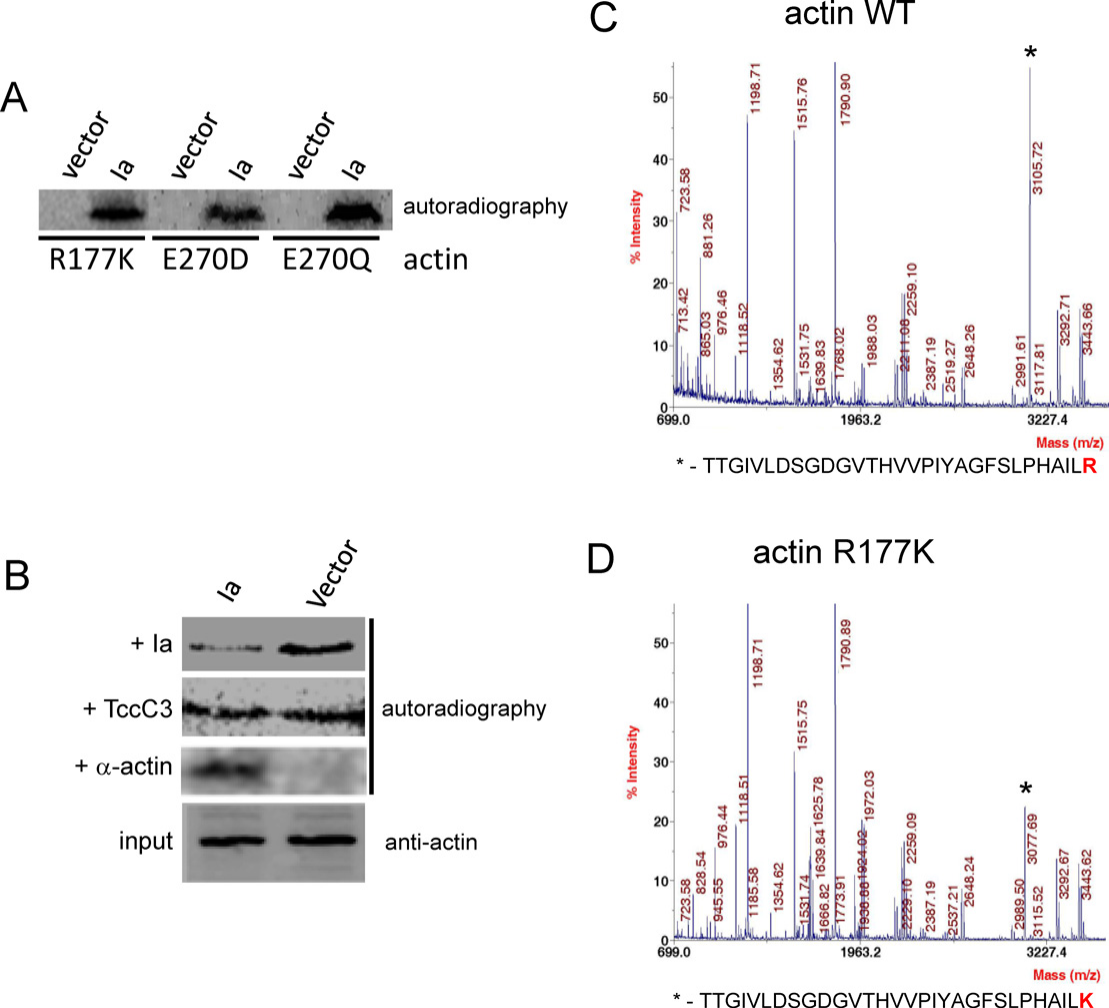
Analysis of Ia production by engineered *S*. *cerevisiae*. (A) Analysis of the synthesis of Ia in *S*. *cerevisiae* strains, producing actin-R177K, E270D and E270Q. Yeast strains, producing actin-R177K, E270D or E270Q and transformed with the Ia-containing plasmid (Ia) or the vector alone (Vector), were cultivated in SGal for 20 h at 30°C. Cells were broken by glass beads treatment and analyzed by ^32^P-ADP-ribosylation in the presence of additionally added purified wild type yeast actin (1 μg). Labeled bands represent modified yeast actin and confirm intracellular production of functionally active Ia by the *S*. *cerevisiae* strains. (B) Production of Ia by the wild type *S*. *cerevisiae* strain. Wild type yeast strains harboring the Ia-containing plasmid (Ia) or the control vector (vector) were cultivated in glucose-containing liquid medium until OD_595_ = 0.5. Afterwards, glucose was replaced by galactose and cultivation continued for 9 h at 30°C. Cells were lysed and the resulting extract preparations were ADP-ribosylated in the presence of Ia (+ Ia), TccC3 toxin of *P*. *luminescens* [42] (+ TccC3), purified muscle actin (+α-actin) or tested in Western blotting with the anti-actin serum to show equal actin concentrations in the samples. (C, D) Mass spectrometry of actin variants. MALDI-TOF MS of wild type (C) and actin-R177K (D) protein variants isolated from *S*. *cerevisiae*. Spectra demonstrate disappearance of R177- and appearance of K177-containing peptide in mass analysis (substituted amino acid residue within identified peptides is shown in red).

To confirm that the observed alterations in yeast growth were indeed linked to Ia production and *ACT1* mutagenesis, we performed additional experiments. Firstly, we prepared extracts from *S*. *cerevisiae*, expressing the actin variants Arg177Lys, Glu270Asp and Glu270Gln, which additionally expressed the enzyme component of iota toxin. These extracts were tested in a ^32^P-ADP-ribosylation assay after addition of purified yeast wild type actin and ^32^P-NAD. Protein separation by SDS-PAGE and autoradiography revealed radiolabeling of actin (~40 kDa) only with yeast transformed with the Ia-coding sequence followed by induction with galactose (Fig 4A). Secondly, we performed a “post-ADP-ribosylation” assay to confirm actin ADP-ribosylation in intact cells. To this end, the *S*. *cerevisiae* strain, producing wild type actin, was transformed with the Ia-expressing plasmid or the vector control and incubated in galactose-containing medium. After 9 h, yeast cells were lysed and used in a ^32^P-ADP-ribosylation assay in the presence of exogenous recombinant Ia purified from *E*. *coli* (“post-ADP-ribosylation”). ADP-ribosylation of intracellular endogenous actin by Ia, which was produced in intact yeast cells, resulted in reduced ^32^P-ADP-ribosylation in the second *in vitro* reaction after addition of recombinant Ia (Fig 4B, upper panel). It should be noted that actin from cell extracts containing intracellularly expressed Ia was readily ADP-ribosylated by *Photorhabdus luminescens* toxin TccC3, which modifies actin at Thr148 [22] independently of an ADP-ribosylation by Ia (Fig 4B). Moreover, addition of muscle actin to the *in vitro* ADP-ribosylation reaction showed radiolabeling of actin with Ia-producing yeast cells but not in control samples, although yeast cell densities in both cultures were similar at this time point (Fig 4B, lower 2 lines). Thirdly, we purified actin from *S*. *cerevisiae* cells, possessing wild type actin or actin-Arg177Lys (i.e. demonstrating Ia-sensitive and Ia-resistant phenotypes, respectively), and subjected the isolated proteins to MALDI-TOF mass spectrometry. Obtained spectra showed the successful substitution of Arg177 by lysine in the corresponding Ia-insensitive strain by disappearance of Arg177- and appearance of Lys177-containing peptides in mass analysis data (Fig 4C and 4D). All these data corroborated our hypothesis that the reduced survival rate of *S*. *cerevisiae* strains is caused by the toxic action of intracellularly produced Ia, while yeast growth in the presence of intracellular Ia is evoked by change of the target Arg177 to lysine and by changes of Glu270 to glutamine and aspartate.

### *In vitro* ADP-ribosylation of actin

**Fig 5:**
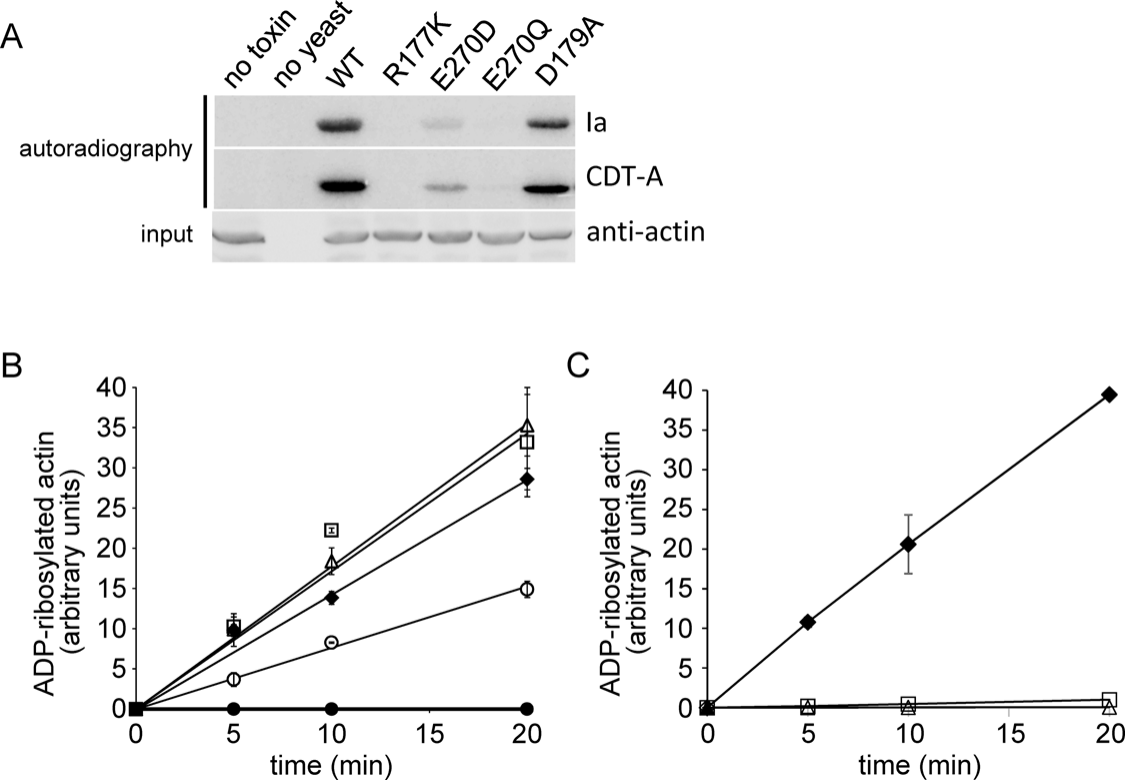
*In vitro* ADP-ribosylation of actin variants produced by *S*. *cerevisiae*. (A) Yeast extracts were prepared from strains producing wild type actin or actin variants with substitutions R177K, D179A, E270D or E270Q and were tested in ^32^P-ADP-ribosylation with Ia or *C*. *difficile* transferase CDT-A. (B) Time course of ^32^P-ADP-ribosylation performed in the presence of Ia with wild-type actin (◆) or actin variants D179E (△), D179A (□), D179K (○) or R177K (●). Means of three measurements with standard deviation are shown. (C) Time course of ^32^P-ADP-ribosylation performed in the presence of Ia with wild type actin (◆) or actin variants E270Q (△), E270D (□). Means of three measurements with standard deviation are shown.

As shown above (Fig 3A), *S*. *cerevisiae* strains, containing actin with substitution of Asp179 (but not Arg177 and less Glu270) were sensitive towards the toxic action of Ia. These results indicated that Ia was able to ADP-ribosylate the corresponding actin variants in the cell. To directly study the substrate properties of engineered actin variants, we employed *in vitro* ^32^P-ADP-ribosylation assays. To this end, we prepared crude cell extracts from *S*. *cerevisiae* strains, producing wild type actin or the actin variants Arg177Lys, Asp179Ala, Glu270Gln and Glu270Asp and tested them in ADP-ribosyltransferase assays with Ia or the highly related enzyme component CDT-A from *C*. *difficile* toxin CDT [23] (as an additional control). As demonstrated in Fig 5A, substitution Asp179Ala did not influence modification of the corresponding actin variants as compared to wild type actin. Whereas actin-Glu270Asp was less efficient modified than wild type actin, the Glu270Gln mutant was a very poor substrate. As expected, mutation of Arg177 made actin completely resistant to the enzymatic action of both Ia and CDT-A.

Next, we isolated the actin mutants by DNAse I affinity chromatography and studied purified actin preparations in a ^32^P-ADP-ribosylation assay. While rates of toxin-catalyzed ADP-ribosylation with the actin variants Asp179Ala or Asp179Glu did not differ from that of wild type actin, Asp179Lys replacement decreased the modification level of the target (Fig 5B). By contrast, the actin variants Glu270Asp and Glu270Gln exhibited very low or only marginal modifications, respectively (Fig 5C).

**Fig 6:**
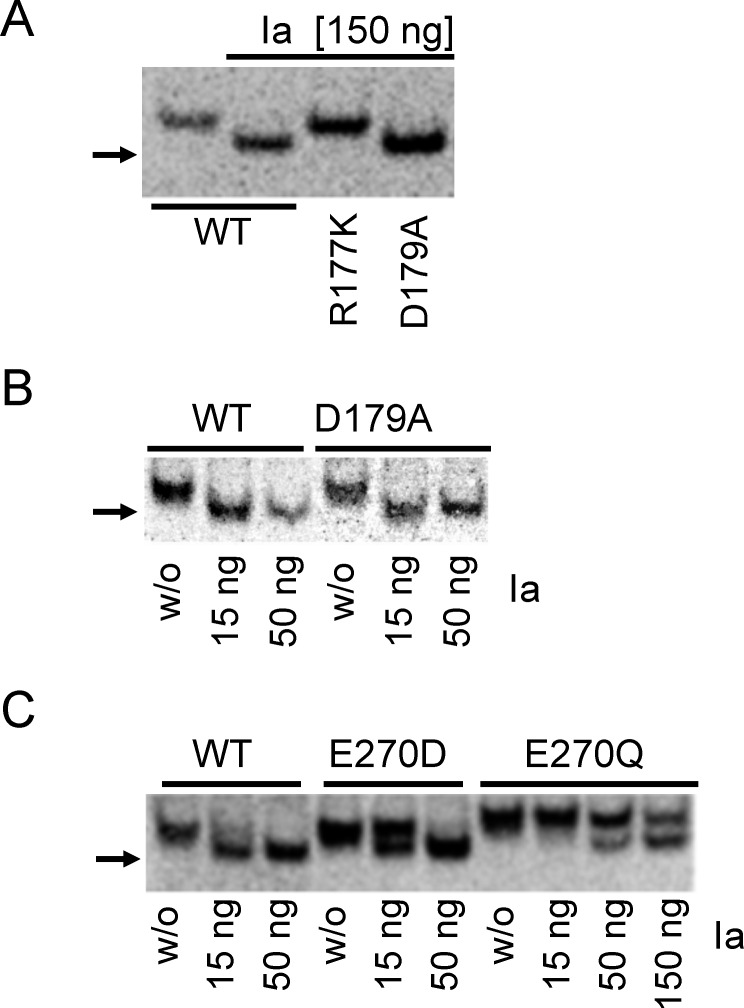
*In vitro* ADP-ribosylation of human β-actin variants. Actin variants were produced in *in vitro* transcription/translation reaction, using as a matrix plasmids coding for human β-actin gene with the corresponding amino acid substitutions (wild type (WT), R177K, D179A, E270D and E270Q). Afterwards, 1 μl of the *in vitro* transcription/translation mix was ADP-ribosylated with Ia (150 ng/10 μl in Panel A; 15 and 50 ng/10 μl in Panel B; and 15, 50 or 150 ng/10 μl in Panel C) or left untreated, without toxin (w/o). Reaction mixes were subjected to non-denaturing polyacrylamide gel electrophoresis and autoradiography (shown) to detect ^35^S-methionine-labelled actin variants. Arrows on the left indicate position of shifted ADP-ribosylated actin.

Moreover, we tested the functional consequences of amino acid exchanges for Ia-induced ADP-ribosylation with human β-actin in an *in vitro* transcription/translation assay. Therefore, we employed native gel electrophoresis that allows separation of ADP-ribosylated actin from non-modified actin by the increased migration velocity of ADP-ribosylated proteins (Fig 6). The studies confirmed that wild type actin and Asp179Ala mutants of human β-actin were readily ADP-ribosylated by Ia (150 ng/ml) under these conditions (Fig 6A). Also at low concentration of Ia (15 ng/ml and 50 ng/ml), Asp179Ala actin was readily modified. The Glu270Asp actin mutant was less modified at low toxin concentrations (15 ng/ml), while most of the protein was shifted at 50 ng/ml (Fig 6C). However, ADP-ribosylation of the Glu270Gln actin mutant was clearly reduced even at high concentration of Ia (150 ng/ml). Thus, the data obtained with human actin confirmed results from studies with yeast actin.

## Discussion

The yeast *S*. *cerevisiae* is a well-established eukaryotic model used to study mechanisms of action of different bacterial products, including effectors of virulence-associated secretion systems and toxins [24–28]. Feasibility of this model relies on high structural conservation of cellular key components targeted by the microbial toxins or effectors [29,30].

Actin is a highly conserved protein. The only actin-coding gene *ACT1* of *S*. *cerevisiae* is located on chromosome VI and is translated into a protein, which is ~87–90% identical to human actin isoforms. This high degree of conservation allows its modification by *C*. *perfringens* iota toxin and suggests the usage of yeast actin as an appropriate model for actin-modifying toxins [31]. At the same time growth behavior of the resulting yeast directly correlates with the functionality of the synthesized proteins. Thus, only functional actin variants produces viable yeast cells. It should be noted that following genetic manipulations only actin variants coded by the transforming plasmids but not the wild type actin is produced by *S*. *cerevisiae*.

We focused on amino acid Asp179 of actin, which is in the vicinity of Arg177, the amino acid target of iota toxin [15]. Tsurumura and coworkers highlighted the possible role of this aspartate residue in the control of the transition state of the Ia-catalyzed ADP-ribosylation reaction. They showed that Asp179 interacts with Arg177 and also proposed that Asp179 has a stabilizing function by contacting the N-ribose moiety of NAD by forming a hydrogen bond [15]. Earlier and in line with this model, Jørgensen *et al*. suggested a substrate-assisted mechanism of ADP-ribosylation of elongation factor eEF2 catalyzed by *Pseudomonas aeruginosa* exotoxin A and diphtheria toxin. In addition to the targeted diphthamide-699 residue of eEF2, the authors stressed the importance of Asp696, which, according to the crystal structures, forms a hydrogen bond with the *N* ribose hydroxyl group during or after ADP-ribosylation of eEF2 [32]. Furthermore, by *in silico* analysis of the region around the acceptor residues of four different ADP-ribosylated targets (Gα_s_ modified at Arg201 by cholera toxin, RhoA modified at Asn41 by *C*. *botulinum* C3 exoenzyme, Gα_i_ ADP-ribosylated at Cys351 by pertussis toxin and actin modified at Arg177 by *C*. *perfringens* iota toxin) they predicted similarly located aspartic/glutamic acids that might react in analogy to the Asp696 of eEF2 (i.e. Glu50, Glu40, Asp350 and Asp179 in Gα_s_, RhoA, Gα_i_ and actin, respectively). However, according to our results, substitution of actin Asp179 by glutamic acid or alanine failed to produce strong effects on the viability of Ia-producing yeast and on ADP-ribosylation of actin. Thus, Asp179 does not seem to play a major role in the Ia-catalyzed ADP-ribosylation of actin. Only change of Asp179 to lysine decreased the ADP-ribosylation of actin and slightly improved yeast survival under Ia gene-inducing conditions. This effect, however, is most likely caused by the introduction of opposing charge, which might interfere with the interaction of actin and Ia involving residues Arg248, Asn255 and Asn256 of the ADP-ribosyltransferase. In this respect, the recent crystal structure analysis of the complex of ADP-ribosyltransferase C3cer with RhoA is of interest [33]. In this study, Glu40 of RhoA was changed to alanine without major effect on ADP-ribosylation, again, indicating that the previous model proposed by Jørgensen et al. has to be modified [32].

Another acidic amino acid residue located near Arg177 of actin is Glu72. Exchange of this residue had no effects on Ia-induced toxicity and ADP-ribosylation (not shown). However, we found that change of Glu270 of actin has major inhibitory effects on toxicity of Ia and toxin-catalyzed ADP-ribosylation. Glu270 is located in the so-called hydrophobic plug, which connects three actin molecules with each other in interstrand interaction of actin filaments [9,34]. Glu270 seems not to be directly involved in the ADP-ribosylation reaction catalyzed by Ia, because the amino acid is too far away from the catalytic site. In the actin-Ia complex, Glu270 is located near the basic amino acid triad of Lys351, Arg352 and Lys353 (KRK motif) of Ia. KRK of Ia is probably an important interaction site, which allows and stabilizes the docking of the toxin to the actin molecule. Therefore, it is plausible that exchange of this residue in actin largely impairs the interaction with iota toxin. In line with a charge dependent effect, we observed that the Ia-induced ADP-ribosylation of the Glu270Gln mutant of actin was stronger inhibited than that of actin Glu270Asp. Both mutations caused only slight reduction in yeast growth. It is worth to note that Glu270 of actin is targeted by the actin-crosslinking domain (ACD) of multifunctional-autoprocessing RTX toxins (MARTX) of *Vibrio cholerae*, *Vibrio vulnificus* and *Aeromonas hydrophila*. The ACDs of these toxins form actin oligomers by crosslinking amino acid Glu270 of one actin molecule with Lys50 of another actin molecule [35–37]. Thus, Glu270 of actin plays a crucial role as a target amino acid for crosslinking MARTX toxins and probably as a docking site for iota toxin.

Taken together, our data indicate that Asp179 of actin plays no major role in iota toxin-catalyzed ADP-ribosylation of actin. Moreover, we identified Glu270 of actin as an essential amino acid, which is most likely involved in the productive interaction of iota toxin with its substrate.

## Materials and Methods

### Strains, vectors and culture conditions

Gene cloning and expression were performed in *Escherichia coli* DH10B (Invitrogen, Grand Island, NY, USA) and BL21 Codon Plus (Stratagene, Santa Clara, CA, USA) strains respectively. Genomic DNA from *S*. *cerevisiae* D273-10B [38] was used for the amplification of the *LEU2* marker gene. *S*. *cerevisiae* MH272–3fα (*ura3*, *leu2*, *his3*, *trp1*, *ade2*) or the diploid MH272–3fα/a (*ura3*/*ura3*, *leu2*/*leu2*, *his3*/*his3*, *trp1*/*trp1*, *ade2*/*ade2*) [39] are the “wild type” strains used in gene engineering work. Plasmids used for cloning of deleting genetic constructs are based on pUC19 vector (New England Biolabs, Frankfurt am Main, Germany) or pBluescript II KS+ (Stratagene). Yeast expression plasmids were constructed using pRS313 [40], YCplac33 [41], YEplac555 [42] and pESC-His (Stratagene) vectors. For Ia production pET28a vector (Novagen, Billerica, MA, USA) was used. A list of used strains, plasmids and PCR primers is presented in Tables A-D in S1 File. Muscle actin was a generous gift from Prof. H.G. Mannherz (Ruhr-University, Bochum, Germany). CDT-A of *C*. *difficile* and TccC3 of *Photorhabdus luminescens* were purified as described previously [22,43].

### Construction of *S*. *cerevisiae* strain containing modified actin

*S*. *cerevisiae* strains were grown on rich medium containing glucose (YPD: 1% yeast extract, 2% peptone, and 2% glucose) or on minimal medium containing 0.67% yeast nitrogen base without amino acids (Difco, Franklin Lakes, NJ, USA) with 2% glucose (SGlc) or 2% galactose (SGal). SGlc and SGal media were enriched with the appropriate supplements (i.e. uracil, leucine, histidine, tryptophan or adenine) based on specific *S*. *cerevisiae* strain requirements. Yeast transformations were performed by the lithium acetate method [44]. To generate functional *LEU2* marker gene used for *ACT1* gene disruption the corresponding coding sequence with minus-312 bp promoter- and plus-293 bp terminator-containing regions was amplified by PCR and cloned into pUC19 vector. *act1*::*LEU2* (i.e. *Δact1*) was constructed by replacing nucleotides from minus-37 to 721 of *ACT1* with functional *LEU2* gene. The deleting construct based on pUC19 vector was delivered into diploid *S*. *cerevisiae* MH272a/α strain, and transformants were selected using minus-leucine minimal medium [29]. Disruptions were confirmed with the primers annealing outside recombination area by PCR with chromosomal DNA isolated from the engineered yeast. Since *act1* deletion is lethal in haploid strain [45], before been sporulated and dissected, the obtained [*ACT1/act1*::*LEU2*] yeast variant was transformed with YCplac33 vector containing *ACT1* gene with the minus-827 promoter- and plus-274 terminator-containing regions. The resulting strain was subsequently sporulated and dissected (Singer Instruments, Somerset, UK). After tetrad analysis a haploid [*Δact1* + YCplac33-*ACT1*] isolate was selected and used for subsequent experiments. YCplac33-*ACT1* was replaced by pRS313-based plasmids encoding different *ACT1* mutants via 5-fluoroorotic acid (5-FOA, Thermo Scientific, Pittsburgh PA, USA) plasmid shuffling method [21].

For site-directed mutagenesis XhoI/KpnI fragment of *ACT1* was cut out from the YCplac33-*ACT1* plasmid and ligated into pBluescript vector. The resulting plasmid was used as a matrix in QuikChange reactions (Promega, Mannheim, Germany) aimed at substitutions of amino acid residues of interest. All mutated genes were initially subcloned back into XhoI/KpnI endonuclease restriction sites of YCplac33-*ACT1* and then transferred *en bloc* with the upstream promoter and downstream terminator regions into pRS313 using EcoRI/SalI sites.

The influence of actin site-substitutions and Ia gene expression upon growth behavior of the resulting yeast variants was analyzed on minimal agar plates by drop-test. To that end, 5-fold serial dilutions of suspensions prepared from overnight agar cultures normalized by OD_595_ measurements were spotted onto agar plates containing the required supplements or YPD. Plates were incubated for 3–4 days at 30°C.

### Cloning of the gene and purification of recombinant Ia of *C*. *perfringens*

The yeast vector for cloning of the Ia-coding gene in *S*. *cerevisiae* (named YEpGal555) was constructed using YEplac555 backbone by introducing GAL1/10 promoter and 3’ untranslated region from pESC-His using SacI/PstI restriction endonucleases with subsequent introduction of NcoI site immediately following BamHI site of the plasmid. A gene coding for the A-subunit of *C*. *perfringens* iota toxin was PCR amplified from chromosomal DNA of *C*. *perfringens* strain E342 (collection of laboratory of molecular pathogenesis, Gamaleya Research Institute, Moscow, Russia) and cloned into YEpGal555 using NcoI/SalI endonuclease sites. For protein production Ia-coding sequence was cut out from the latter plasmid with BamHI/SalI restriction endonucleases and ligated into pET28a.

For protein production *E*. *coli* BL-21 Codon Plus strain transformed with Ia-coding gene within pET28a plasmid was grown on a shaker at 37°C until OD_595_ = 0.8. Expression of *ia* gene was induced by 1 mM IPTG overnight at 28°C. Cells were collected by centrifugation and suspended in a 6xHis-binding buffer (20 mM phosphate buffer (pH 7.4), 0.5 M NaCl, and 5 mM imidazole) supplemented with 1 mM PMSF and protease inhibitor cocktail (Roche, Mannheim, Germany). Bacteria were lysed by sonication. Bacterial extract, clarified by centrifugation, was loaded onto a column with Ni-IDA resin (Macherey-Nagel, Duren, Germany). The column was washed sequentially with 6xHis-binding buffer and with 6xHis-binding buffer plus 30 mM imidazole. Ia-containing preparation was eluted with 500 mM imidazole and dialyzed overnight against 20 mM Tris-HCl (pH 7.4) with 50 mM NaCl. After dialysis, the sample was applied onto Mono Q 5/50 GL (GE Healthcare, Pittsburgh, PA, USA) column. Flow-through fraction, containing Ia, was collected, concentrated and subjected to gel chromatography on Superdex 75 10/300 GL (GE Healthcare) equilibrated with TBS (20 mM Tris-HCl (pH 7.4) and 150 mM NaCl).

### Isolation of yeast actin

Purification of yeast actin was performed according to the DNase I affinity method originally described by Kron *et al* [46] with modifications by Goode [47]. To this end, yeast cells were grown for 24 h in YPD-medium till OD_595_ = 5–7, collected by centrifugation, resuspended in a small amount of lysis buffer (20 mM Tris-HCl (pH 7.4), 150 mM NaCl, 2 mM DTT, 1 mM PMSF and protease inhibitor cocktail) and frozen drop-wise in liquid nitrogen. Frozen yeast cells were lysed using Mixer Mill MM400 device (Retsch, Haan, Germany). Obtained yeast powder was resuspended in a lysis buffer, cleared by centrifugation and applied onto a column, containing DNase I (Sigma) immobilized on Affi-Gel 10 (Bio-Rad) according to the manufacturer’s protocol. After application of yeast extract, the column was washed with G-buffer (20 mM Tris-HCl (pH 7.4), 0.5 mM ATP, 0.2 mM DTT and 0.2 mM CaCl_2_) and wash buffer (G-buffer with 10% formamide). Actin was eluted with 50% formamide in G-buffer. The protein-containing fractions were immediately diluted 5-fold with G-buffer and dialyzed against it overnight. Afterwards, samples were concentrated using Vivaspin 20 (Sartorius, Goettingen, Germany), dispensed into aliquots, frozen in liquid nitrogen and stored at -80°C until use.

Mass-analysis of purified wild type actin and actin R177K variant was performed on 4800 MALDI TOF/TOF Analyzer (AB Sciex GmbH, Darmstadt, Germany) at TOPLAB GmbH, (Martinsried, Germany).

### *In vitro* ADP-ribosylation

Actin modification *in vitro* was performed either with crude yeast lysate (2.5 mg/ml) or with purified actin (100 μg/ml) by recombinant Ia or CDT-A (0.25 μg/ml of Ia or CDT-A for crude yeast extract and 1 μg/ml of Ia for the purified protein) in a total volume of 10 μl. Reaction mixtures contained 1 μM non-radiolabeled NAD, 0.1 μCi ^32^P-NAD (Perkin Elmer, Hamburg, Germany), 0.5 mM DTT, 20 mM Hepes-KOH (pH 7.4), 100 mM KCl and 2 mM MgCl_2_ (ADP-ribosylation with yeast extract) or 1 μM non-radiolabeled NAD, 0.1 μCi ^32^P-NAD, 20 mM Tris-HCl (pH 7.4), 0.5 mM ATP, 0.2 mM DTT and 0.2 mM CaCl_2_ (ADP-ribosylation with purified actin). Incubation was at 30°C for the indicated times. Reactions were stopped by the addition of Laemmli sample buffer and heating at 95°C for 5 min. Then, the samples were subjected to SDS-PAGE and autoradiography using Storm 820 phosphorimager (GE Healthcare). Autoradiograms were quantified by ImageQuant 5.2 (Molecular Dynamics, Vienna, Austria).

Point mutations in human β-actin gene, resulting in actin variants with Arg177Lys, Asp179Ala, Glu270Asp and Glu270Gln substitutions, were produced by QuikChange reaction using the matrix plasmid β-actin-pET28a [22] and the corresponding primers (Table C in S1 File). Engineered actin variants were synthesized in *in vitro* transcription/translation reaction (TNT Coupled Reticulocyte Lysate System, Promega) using the plasmids, coding for the proteins with the amino acid substitutions, and ^35^S-methionine (specific activity, 37 TBq/mmol; Hartmann Analytic, Braunschweig, Germany). Afterwards, 1 μl of the *in vitro* transcription/translation mix, containing co-translationally ^35^S-labeled actin, was ADP-ribosylated with 0.2 mM NAD in 20 mM Tris-HCl (pH 7.4), 0.5 mM ATP, 0.2 mM DTT and 0.2 mM CaCl_2_ and various concentrations of Ia and subjected to polyacrylamide gel electrophoresis under non-denaturing conditions [22]. Finally, gels were processed by autoradiography (Storm 820 phosphorimager).

### Preparation and analysis of yeast extracts

Crude yeast extracts for Western blotting and enzymatic assays were prepared by glass-beads disruption in 20 mM Hepes-KOH (pH 7.4) with 100 mM KCl, 2 mM MgCl_2_, 0.5 mM DTT, 1 mM PMSF and protease inhibitor cocktail. Yeast extracts and purified proteins were analyzed by polyacrylamide gel electrophoresis in sodium dodecylsulfate buffer and Western blotting. Protein concentrations were estimated using Coomassie Brilliant Blue G-250 dye. For actin immunodetection monoclonal antibodies (mAbGEa, Novus Biologicals, Cambridge, United Kingdom) were used at 1/2000 dilution.

## Supporting Information

S1 Fig PCR confirmation of chromosomal ACT1 deletion. (TIFF)

S2 Fig Genetic analysis of engineered *S*. *cerevisiae* A/alpha *ACT1/act1*::*LEU2* strain. (TIFF)

S1 File Tables of strains (Table A), plasmids (Table B), primers used for gene cloning/mutagenesis (Table C) and primers used in PCR analysis of *S*. *cerevisiae* deletion mutants (Table D) (DOCX)

S1 Legends (DOCX)

S1 Results (DOCX)
