# Supplementary figures and images for: Roles of Asp179 and Glu270 in ADP-Ribosylation of Actin by Clostridium perfringens Iota Toxin
Source: PLoS One. 2015 Dec 29;10(12):e0145708. doi: 10.1371/journal.pone.0145708 (PMC4699905; doi:10.1371/journal.pone.0145708)

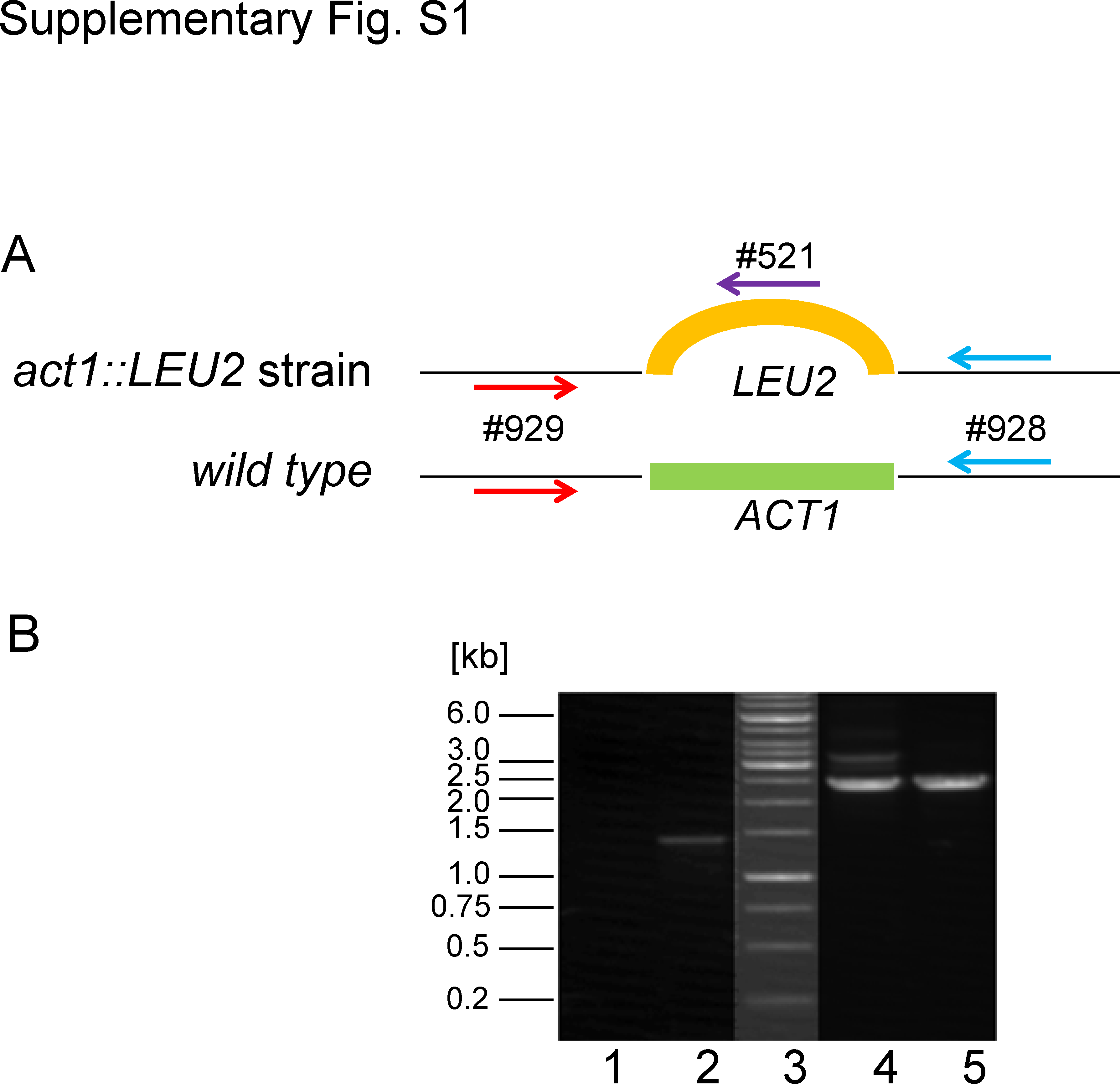

Supplement: S1 Fig — (TIFF) [file pone.0145708.s001.tiff]

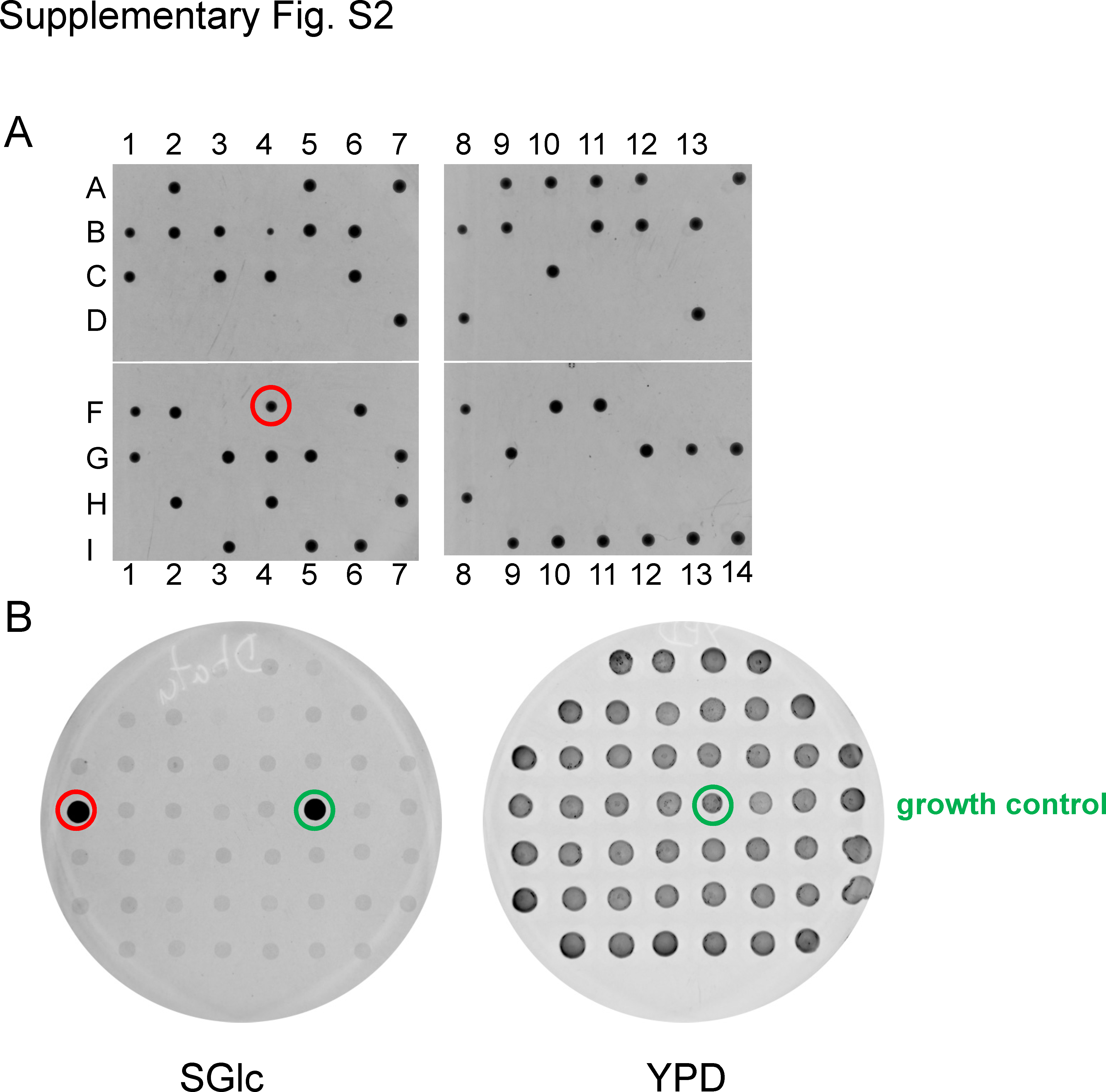

Supplement: S2 Fig — (TIFF) [file pone.0145708.s002.tiff]
