# Supplementary material for: Roles of Asp179 and Glu270 in ADP-Ribosylation of Actin by Clostridium perfringens Iota Toxin
Source: PLoS One. 2015 Dec 29;10(12):e0145708. doi: 10.1371/journal.pone.0145708 (PMC4699905; doi:10.1371/journal.pone.0145708)
Supplement: S1 File — (DOCX) [file pone.0145708.s003.docx]

**Supplemental Information**

**S1 File**

**Table A. Strains**

| Strain/plasmid | Description/genotype | Source/citation |
| --- | --- | --- |
| *E. coli* DH10B | F– mcrA Δ(mrr-hsdRMS-mcrBC) Φ80lacZΔM15 ΔlacX74 recA1 endA1 araD139 Δ(ara leu) 7697 galU galK rpsL nupG λ– | Invitrogen |
| *E. coli* BL21-CodonPlus(DE3)-RIL strain | *F– ompT hsdS(rB– mB–) dcm+ Tetr gal endA Hte [argU ileY leuW Camr]* | Stratagene |
| *S. cerevisiae* MH272-3fa/α | *ura3/ura3, leu2/leu2, his3/his3, trp1/trp1, ade2/ade2* | [39] |
| *S. cerevisiae* MH272-3fα | *ura3, leu2, his3, trp1, ade2* | [39] |
| *S. cerevisiae* D273-10B | *MAT*α *mal [rho+]* | [38] |
| *S. cerevisiae* SC 475 | A/α ACT1/act1::LEU2 | This study |
| *S. cerevisiae* SC 481 | A/α *ACT1/act1::LEU2* + *ACT1*[Ura3] | This study |
| *S. cerevisiae* SC 483 | haploid *act1::LEU2* + *ACT1*[Ura3] | This study |
| *S. cerevisiae* SC 489 | haploid *act1::LEU2* + *ACT1*[His3] | This study |
| *S. cerevisiae* SC 495 | haploid *act1::LEU2* + *ACT1* R177K[His3] | This study |
| *S. cerevisiae* SC 501 | haploid *act1::LEU2* + *ACT1* D179E[His3] | This study |
| *S. cerevisiae* SC 502 | haploid *act1::LEU2* + *ACT1* D179A[His3] | This study |
| *S. cerevisiae* SC 501 | haploid *act1::LEU2* + *ACT1* D179E[His3] | This study |
| *S. cerevisiae* SC 564 | haploid *act1::LEU2* + *ACT1* E72A[His3] | This study |
| *S. cerevisiae* SC 625 | haploid *act1::LEU2* + *ACT1* E270D[His3] | This study |
| *S. cerevisiae* SC 626 | haploid *act1::LEU2* + *ACT1* D270Q[His3] | This study |

**Table B. Plasmids**

| Plasmid/vector | Description | Source/citation |
| --- | --- | --- |
| pUC19 | *E. coli* general cloning vector (Ap) | NEB |
| pBluescript II KS+ | *E. coli* general cloning vector (Ap) | Stratagene |
| pET28 | *E. coli* expression vector (Kn) | Novagen, |
| pRS313 | *E. coli*/*S. cerevisiae* shuttle vector [HIS3] | [40] |
| pESC-His | *E. coli*/*S. cerevisiae* shuttle vector [HIS3] | Stratagene |
| YEplac555 | *E. coli*/*S. cerevisiae* shuttle vector [ADE2] | [42] |
| YCplac33 | *E. coli*/*S. cerevisiae* shuttle vector [URA3] | [41] |
| YEpGal555 | *E. coli*/*S. cerevisiae* shuttle vector [ADE2] | This study |
| p1177 | *ACT1* in YCplac33 | This study |
| p1182 | *ACT1* in pRS313 | This study |
| p1242 | *ACT1*-knock-outing construct in pUC19 | This study |
| p1279 | *ACT1* R177K in pRS313 | This study |
| p1296 | *ACT1* D179E in pRS313 | This study |
| p1297 | *ACT1* D179A in pRS313 | This study |
| p1353 | *ACT1* D179K in pRS313 | This study |
| p1377 | *ACT1* E72A in pRS313 | This study |
| p1323 | *ia* in YEpGal555 | This study |
| p1330 | *ia* in pET28a | This study |
| p1587 | *ACT1* E270D in pRS313 | This study |
| p1588 | *ACT1* E270Q in pRS313 | This study |
| p1566 | Human β-actin in pET28 | This study |
| p1567 | Human β-actin R177K in pET28 | This study |
| p1568 | Human β-actin D179A in pET28 | This study |
| p1640 | Human β-actin E270Q in pET28 | This study |
| p1639 | Human β-actin E270D in pET28 | This study |

**Table C. Primers used for gene cloning/mutagenesis**

| ID number | Nucleotide sequence (5’-to-3’) | Resulting plasmid/construct |
| --- | --- | --- |
| # 501 | CACAG***GAGCTC***TATCGCACAGAATC | p1242, *LEU2* |
| # 502 | ATTA***GGATCC***ATAGTTTCATGATTTTC | p1242, *LEU2* |
| # 925 | CTTCCTTATC***GAATTC***TCAAAACC | p1242, p1182, *ACT1* |
| # 926 | ATAACCAAAGCAGC***GAGCTC***TAAAC | p1242, *ACT1* |
| # 951 | TAACGAA***GGATCC***AGAGCCCCAGA | p1242, *ACT1* |
| # 928 | GATACACG***GTCGAC***TGGATAAAC | p1242, p1182, *ACT1* |
| # 1019 | GATTTACC***CATGGC***AAGCAATTATATTAC | p1330, *ia* |
| # 1020 | GATAACG***GTCGAC***TTATCATAGCTG | p1330, *ia* |
| # 986 | CACGCCATTTTGAAAATCGATTTGG | p1279, *ACT1* R177K |
| # 987 | CCAAATCGATTTTCAAAATGGCGTG | p1279, *ACT1* R177K |
| # 1006 | TTGAGAATCGAATTGGCCGGTAGAG | p1296, *ACT1* D179E |
| # 1007 | CTCTACCGGCCAATTCGATTCTCAA | p1296, *ACT1* D179E |
| # 1008 | TTGAGAATCGCTTTGGCCGGTAGAG | p1297, *ACT1* D179A |
| # 1009 | CTCTACCGGCCAAAGCGATTCTCAA | p1297, *ACT1* D179A |
| # 1026 | CCATTTTGAGAATCAAATTGGCCGG | p1353, *ACT1* D179K |
| # 1027 | CCGGCCAATTTGATTCTCAAAATGG | p1353, *ACT1* D179K |
| # 1044 | TTACCCAATTGCACACGGTATTG | p1377, *ACT1* E72A |
| # 1045 | CAATACCGTGTGCAATTGGGTAA | p1377, *ACT1* E72A |
| # 1251 | TTTGGGTTTGGATTCTGCCGGTAT | p1587, *ACT1* E270D |
| # 1252 | ATACCGGCAGAATCCAAACCCAAA | p1587, *ACT1* E270D |
| # 1253 | GTTTTGGGTTTGCAATCTGCCGGT | p1588, *ACT1* E270Q |
| # 1254 | GTTTTGGGTTTGCAATCTGCCGGT | p1588, *ACT1* E270Q |
| # 1278 | CTGGGCATGCAGTCCTGTGGCAT | p1640, human β-actin E270Q |
| # 1279 | ATGCCACAGGACTGCATGCCCAG | p1640, human β-actin E270Q |
| # 1227 | CATGCCATCCTGAAGCTGGACCTGG | p1567, human β-actin R177K |
| # 1228 | CCAGGTCCAGCTTCAGGATGGCATG | p1567, human β-actin R177K |
| # 1229 | CTGCGTCTGGCCCTGGCTGGC | p1568, human β-actin D179A |
| #1230 | GCCAGCCAGGGCCAGACGCAG | p1568, human β-actin D179A |
| # 1276 | CTGGGCATGGATTCCTGTGGCAT | p1639, human β-actin E270D |
| # 1277 | ATGCCACAGGAATCCATGCCCAG | p1639, human β-actin E270D |

Engineered restriction endonuclease sites (if any) are shown in bold and underlined font. First oligonucleotide in a pair for the corresponding plasmid is sense, second – antisense.

**Table D. Primers used in PCR analysis of *S. cerevisiae* deletion mutants**

| ID number | Nucleotide sequence (5’-to-3’) | Target gene |
| --- | --- | --- |
| # 929 | TGAACGAAACCACTCAGAAG | 1112 bp upstream of *ACT1* start codon |
| # 521 | CGGCTGTGATTTCTTGACC | *LEU2* |
| # 928 | GATACACGGTCGACTGGATAAAC | 289 bp downstream from *ACT1* stop codon |
