# Supplementary material for: Roles of Asp179 and Glu270 in ADP-Ribosylation of Actin by Clostridium perfringens Iota Toxin
Source: PLoS One. 2015 Dec 29;10(12):e0145708. doi: 10.1371/journal.pone.0145708 (PMC4699905; doi:10.1371/journal.pone.0145708)
Supplement: S1 Legends — (DOCX) [file pone.0145708.s004.docx]

**Supplemental Information**

**S1 Legends**

**S1 Fig. PCR confirmation of chromosomal *ACT1* deletion.**

(**A**) Schematic illustration of the positions of PCR primers used for the verification of knock-out results.

(**B**) Agarose gel analysis of products from PCR reactions performed with primers #929/#521 on DNA isolated from wild type yeast (*lane 1*) and *ACT1*-knock-outed yeast (*lane 2*). Products from PCR reactions with the primers #929/#928 on DNA isolated from *ACT1*-knock-outed yeast (*lane 4*) and wild type yeast (*lane 5*). Marker DNA size in kilobase pairs is shown on the left. Please note appearance of ~1.4 kb fragment (*lane 2*) and additional ~3.2 kb fragment (*lane 4*) confirming correct insertion of the marker in knock-out *act1::LEU2* strain.

**S2 Fig. Genetic analysis of engineered *S. cerevisiae* A/alpha *ACT1/act1::LEU2* strain.**

(**A**) Spore tetrad dissection of the engineered yeast strains. Sporulation was induced by cultivation of yeast on presporulation and minimal sporulation agar media [53] followed by tetrad dissection. Each spore from a tetrad was inoculated in position ABCD or FGHI. Distribution 2/2 of live/dead yeast cells suggests the destruction of essential genes within a single allele. A colony arisen from erroneously taken vegetative yeast cell is encircled in red.

(**B**) Marker analysis of the resulting haploid yeast cells. Forty seven spores, outgrown on YPD plate, were transferred onto glucose containing agar medium (SGlc-medium) supplemented with uracil, histidine, tryptophan and adenine (*left panel*) or YPD agar (*right panel*). No viable colonies appeared on minus-leucine media, suggesting an insertion of the *LEU2* auxotrophy marker into the essential *Act1* gene. A colony, arisen from erroneously taken vegetative yeast cell is encircled in red. A control colony (growth control), demonstrating marker requirements of the original diploid strain (*S. cerevisiae* A/alpha *ACT1/act1::LEU2*) is encircled in green.
