# Supplementary material for: Roles of Asp179 and Glu270 in ADP-Ribosylation of Actin by Clostridium perfringens Iota Toxin
Source: PLoS One. 2015 Dec 29;10(12):e0145708. doi: 10.1371/journal.pone.0145708 (PMC4699905; doi:10.1371/journal.pone.0145708)
Supplement: S1 Results — (DOCX) [file pone.0145708.s005.docx]

**Supplemental Information**

**S1 Results**

Construction of *S. cerevisiae* *Δact1* variant

In order to analyze the importance of amino acid residues in actin on ADP-ribosylation by Ia we engineered an *ACT1*-deficient diploid yeast variant. During these experiments the 758 nucleotides of the sequence coding for chromosomal *ACT1* were substituted by *LEU2* auxotrophic marker, which inactivated the corresponding gene of one allele, leaving second allele of the gene intact. Actin represents an essential molecule. Viable yeast cells should always produce a functional copy of the protein. Thus, to rescue mutated yeast lacking chromosomal *ACT1*, engineered diploid strain *S. cerevisiae* [*ACT1/act1::LEU2*] was transformed with the plasmid, containing the corresponding gene under the control of its own promoter and uracil marker (*URA3)*. The resulting yeast variant was then subjected to sporulation and dissection. Tetrad analysis allowed selection of a strain *S. cerevisiae* [*act1::LEU2*] + YEpGal555-*ACT1* [*URA3*], which was used subsequently as a background variant for further actin substitutions.

This strain was transformed with plasmids coding for different actin variants and containing *HIS3* as a metabolic marker. Subsequently, we used the 5-FOA shuffling method, which allows the elimination of *URA3-*containing plasmid, leaving at the same time a functional plasmid construct with *HIS3* marker within yeast cell [46]. Thereby, we deleted the native wild type *ACT1* gene and replaced it by mutated versions, resulting in synthesis of actin molecules with substitutions of R177, D179 and E270.

Accuracy of performed deletion procedure was determined in special experiments as follows. Correct positions of inserted marker genes were confirmed by PCR with the primers annealing outside recombination area and within the *LEU2* gene (S1 Fig). Next, as an additional proof of precision of our deletion experiments, the resulting diploid engineered strain *S. cerevisiae* [*ACT1/act1::LEU2*] was sporulated, tetrads were dissected and obtained colonies were studied for their marker requirements (S2 Fig). Finally, actin purified from *S. cerevisiae* containing wild type actin or actin with R177K substitution, was analyzed by MALDI-TOF mass spectrometry.

The results of all these experiments demonstrated precise incorporation of used markers into targeted loci of the chromosome, destruction of the chromosomal sequence coding for yeast wild-type actin and substitution of the wild type actin by actin variants with certain amino acid residue replacements.
